# A 2-*Cys peroxiredoxin* gene from *Tamarix hispida* improved salt stress tolerance in plants

**DOI:** 10.1186/s12870-020-02562-6

**Published:** 2020-07-30

**Authors:** Yuanyuan Wang, Zhongyuan Liu, Peilong Wang, Bo Jiang, Xiaojin Lei, Jing Wu, Wenfang Dong, Caiqiu Gao

**Affiliations:** State Key Laboratory of Tree Genetics and Breeding (Northeast Forestry University), Harbin, 150040 China

**Keywords:** Salt stress, *Tamarix hispida*, *Th2CysPrx*

## Abstract

**Background:**

Peroxiredoxins (Prxs) are a large family of antioxidant enzymes that respond to biotic and abiotic stress by decomposing reactive oxygen species (ROS). In this study, the stress tolerance function of the *Th2CysPrx* gene was further analysed. It lays a foundation for further studies on the salt tolerance molecular mechanism of *T. hispida* and improved salt tolerance via transgenic plants.

**Results:**

In this study, the stress tolerance function of the *Th2CysPrx* gene was further analysed. The results of transgenic tobacco showed higher seed germination rates, root lengths, and fresh weight under salt stress than wild-type tobacco. Simultaneously, physiological indicators of transgenic tobacco and *T. hispida* showed that *Th2CysPrx* improved the activities of antioxidant enzymes and enhanced ROS removal ability to decrease cellular damage under salt stress. Moreover, *Th2CysPrx* improved the expression levels of four antioxidant genes (*ThGSTZ1*, *ThGPX*, *ThSOD* and *ThPOD*).

**Conclusions:**

Overall, these results suggested that *Th2CysPrx* enhanced the salt tolerance of the transgenic plants. These findings lay a foundation for further studies on the salt tolerance molecular mechanism of *T. hispida* and improved salt tolerance via transgenic plants.

## Background

Abiotic stresses, such as drought, salinity, and extreme temperature, among others, negatively affect the growth and yield of plants, resulting in very large economic losses. Among the harmful environmental stresses, salt stress in particular leads to slower plant growth and declines in cultivated plant production [1]. In the long-term evolutionary process, some plants, such as *Atriplex canescens* [2], *Halostachys caspica* [3], *Suaeda salsa* [4], and *Salicornia brachiata* [5], among others, have gradually adapted to a salt stress environment and can grow in dry and saline land.

*Tamarix hispida* is a typical woody halophyte that can form natural forests in saline alkali soil with 1% salt content. In addition, it can also endure drought stress, which makes it an ideal material to clone genes related to drought and salt tolerance and to study the salt tolerance mechanism of woody halophytes [6]. Many previous studies have been conducted to examine the mechanism of salt and drought tolerance and the function of stress resistance genes of *T. hispida*. For example, *TheIF1A*, *ThDREB*, *ThZFP1*, *ThGSTZ1* and *ThPOD3* in *T. hispida* increase salt and drought tolerance by regulating superoxide dismutase (SOD) and peroxidase (POD) activities to reduce reactive oxygen species (ROS) accumulation [7–11]. These results all suggest that scavenging of ROS plays an important role in the response of *T. hispida* to salt stress.

*POD*, *SOD*, *CAT* (*Catalase*), *GPX* (*Glutathione peroxidase*) and *GST* (*Glutathione S-transferases*) are important ROS-scavenging genes. Gao et al. [12] found that *ThGSTZ1* improved tolerance to abscisic acid ABA and methyl viologen (MV) stress by augmenting the activities and expression levels of *ThGPX*, *ThSOD*, and *ThPOD* and the ROS-scavenging capacity. The *NtSOD*, *NtAPX*, *NtCAT*, *NtPOX*, and *NtGST* genes play roles in eliminating ROS and increasing stress tolerance in plant under stress. Fortunately, overexpressed wheat *TaWRKY44* was found to activate the above five genes [13]. In *Betula platyphylla*, *BplMYB46* could increase the ROS-scavenging capacity and proline content to improve salt and osmotic tolerance by affecting the expression of genes, including *POD*, *SOD* and *P5CS* (Δ1-pyrroline-5-carboxylate synthetase) [14]. Zhang et al. [15] found that under NaCl stress, ALA in tomatoes increased the activity of ROS-scavenging antioxidant enzymes and the expression of the *SOD*, *APX* and *POD* genes encoding these enzymes.

Peroxiredoxins (Prxs) are a family of non-haem peroxidases that are widely present in animals, plants and microorganisms. The biological function of these peroxidases is to regulate the balance of ROS and intracellular signal transduction through hydrogen peroxide (H_2_O_2_), alkyl hydroperoxides, and peroxynitrite [16–19]. In plants, based on the number and position of conserved Cys residues, Prxs are grouped into four classes: 1CysPrx, 2CysPrx, type-II Prx and PrxQ. The 1CysPrxs have only one conserved Cys residue, and PrxQ contains two cysteine residues that are catalytically active and linked by intramolecular disulphide bonds [20]. Both 2-Cys Prx and type-II Prx have two conserved Cys residues, but the difference between them is that 2-Cys Prx is a stromal protein [21], and Prx-II has various isoforms [22].

Prxs operate in a particular way during plant growth, development and stress tolerance. For example, Kim et al. [23] found that *2CysPrxs* could eliminate H_2_O_2_ by participating in an alternative water-water cycle and protect photosynthetic structures against oxidative damage under environmental restriction. Pea chloroplast *2CysPrx* and mitochondrial *Prx IIF* affect the structure and peroxidase activity of photosynthetic structures [24]. Kim et al. [25] found that *2CysPrx* from *Oryza sativa* could increase tolerance to ROS-induced oxidative stress by improving cellular redox homeostasis. Overexpression of *2CysPrx* in tall fescue plants increases resistance to oxidative stress and antioxidant activity [26]. Prx also detoxifies ROS and modulates signalling responses [27]. Interestingly, there is no direct evidence that the *Prxs* gene of *T. hispida* is involved in ROS scavenging and the abiotic stress response. In a previous study, Gao et al. [28] cloned four *Prxs* genes from *T. hispida*. Real-time quantitative PCR (qRT-PCR) analysis indicated that these genes could respond to several abiotic stresses and ABA application. However, *Th2CysPrx* displayed a unique expression pattern under the studied stress conditions. In the present study, the role of *Th2CysPrx* in salt stress was further demonstrated, and elucidated the molecular mechanism of this gene under salt stress. It also provided potential application prospects for molecular breeding to improve salt tolerance.

## Results

### Overexpression of *Th2CysPrx* improves salt stress tolerance in transgenic tobacco

**Fig. 1:**
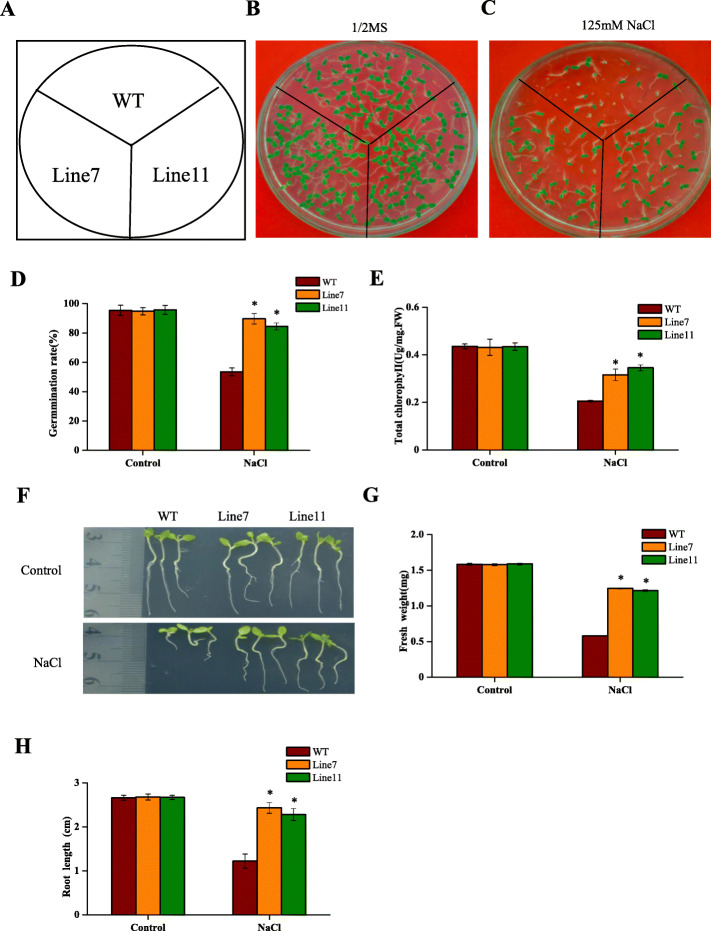
Growth of *Th2CysPrx* transgenic lines and WT tobacco under NaCl stress. **a** The distribution chart of the WT, Line7 and 11 in the plates. **b**, **c** Seed germination of *Th2CysPrx*-transformed lines and WT plants under normal conditions (1/2 MS) and salt (125 mM NaCl) stress for 10 d. **d** Seed germination rates assay. **e** Chlorophyll contents assay. **f** The growth phenotype of *Th2CysPrx* transformed and WT plants. Analysis of fresh weight (**g**) and root length (**h**). Control: under normal conditions. All experiments were repeated three times. Data are the mean ± SD of three independent experiments. Asterisks (*, *P* < 0.05) indicate significant difference compared with WT plants

To study the biological role of *Th2CysPrx* in the salt stress response, the 14 resistant lines were obtained. The qRT-PCR results showed that *Th2CysPrx* gene were overexpressed in all 14 lines (Additional file 1: Figure S1). Two representative T_3_ homozygous transgenic lines (Line 7 and Line 11) were randomly selected for further salt tolerance analysis. The germination and growth of transgenic and wild-type (WT) tobacco were compared during exposure to normal and 125 mM NaCl stress conditions. The results showed that, under normal conditions, there was no obvious difference in the germination rates and seedling growth between transgenic and WT plants (Fig. 1a-c). Under salt stress, however, the germination rate and seedling growth of transgenic plants was significantly better than those of WT plants. Under NaCl stress, the germination rates of transgenic lines were 89.7% (Line 7) and 84.5% (Line 11), while that of WT plants was only 53.5% (Fig. 1d). The chlorophyll content of the transgenic lines under NaCl treatment was 1.54- and 1.68-fold greater, respectively, than that of the WT plants (Fig. 1e). The average fresh weight of the transgenic lines under NaCl treatment was 2.15- and 2.10-fold greater, respectively, than that of the WT plants (Fig. 1g). The average root length of transgenic lines under NaCl treatment was 1.99- and 1.87-fold greater, respectively, than that of WT plants (Fig. 1h). It was apparent that salt stress significantly inhibited the growth of transgenic and WT plants. However, the growth of transgenic plants was significantly superior to that of WT plants. These results indicated that the expression of *Th2CysPrx* in tobacco could significantly increase the salt tolerance of the plants.

### The *Th2CysPrx* gene significantly improves ROS scavenging and reduces cell damage in transgenic tobacco

**Fig. 2:**
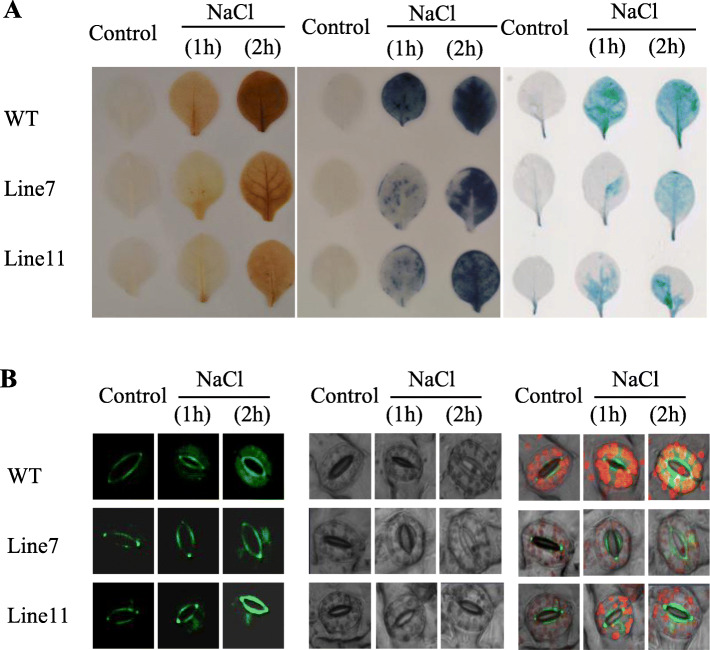
Under 200 mM NaCl stress, histochemical staining analysis of ROS accumulation and cell damage of transgenic lines and WT plants in 2-month-old. **a** Diaminobenzidine (DAB) and nitroblue tetrazolium (NBT) revealed the accumulation of O_2_^−^ and H_2_O_2_ in the leaves of transgenic (Line7 and 11) and (WT) plants subjected to salt stress, Evans Blue staining analysis of cell death. **b** H_2_DCF-DA staining of guard cells. All experiments were repeated three times

The H_2_O_2_ and O_2_^−^ concentration in the transgenic lines and WT plants were examined by 3,3′-diaminobenzidine (DAB) and nitro blue tetrazolium (NBT) staining, respectively. The results showed that under normal condition, there was no significant variation in the ROS generated by the three lines. In contrast, under salt stress, the WT plants exhibited deeper staining, suggesting much greater ROS accumulation in the WT than the transgenic lines under stress conditions (Fig. 2a). 2,7-dichlorofluorescin diacetate (H_2_DCF-DA) staining of the leaves of the transgenic and WT lines was also performed after 1 and 2 h of salt stress, respectively. There were no obvious differences in ROS levels in intact guard cells under normal conditions. However, after salt stress, the WT plants showed increased ROS accumulation in guard cells compared with the transgenic lines (Fig. 2b). The transgenic lines exhibited lower ROS content than the WT plants. These results indicated that *Th2CysPrx* led to a significant reduction of ROS accumulation in plant cells under salt stress.

**Fig. 3:**
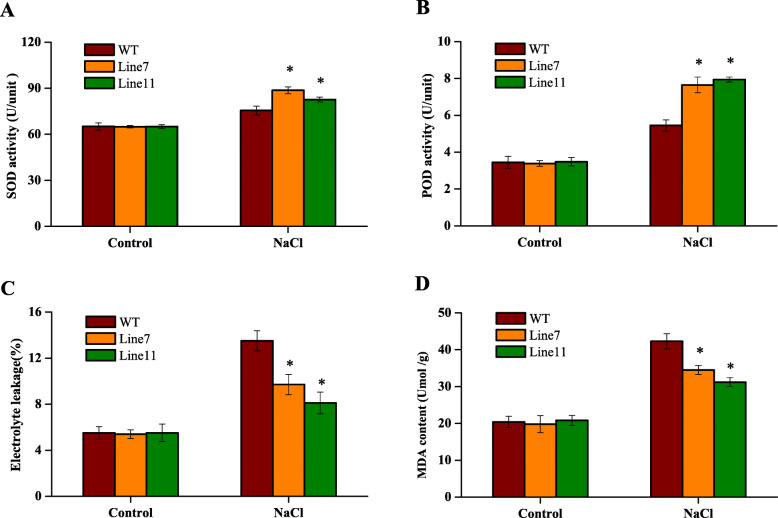
Physiological index analysis of 2-month-old seedlings of *35S::Th2cys* transgenic and WT plants under salt conditions. **a** Chlorophyll contents assay. **b** SOD activity. **c** POD activity. **d** MDA content. **e** Electrolyte leakages. Normally watered plants were used as controls. CK: Tobacco plants transformed with empty pROKII; *35::Th2Cys*: Tobacco overexpressing *Th2CysPrx*; All experiments were repeated three times. Data are the mean ± SD of three independent experiments. Asterisks (*, *P* < 0.05) indicate significant difference compared with WT plants

Furthermore, SOD and POD activities in the transgenic and WT plant lines were measured under salt stress. The results revealed no significant differences between two independent transgenic lines and WT plants under normal conditions. In contrast, SOD and POD activities were higher in the two transgenic lines than in the WT plants under NaCl stress. Specifically, the SOD activities of the transgenic lines were 1.17- and 1.10-fold higher than that of WT (Fig. 3a). And the POD activity values in the transgenic lines were 1.40- and 1.46-fold higher, respectively, than those in the WT plants (Fig. 3b).

Similarly, there were no obvious differences between the transgenic and WT plant lines based on Evans blue staining, malondialdehyde (MDA) contents and electrolyte leakage (EL) rates under normal conditions. Under salt stress conditions, deeper Evans blue staining was observed, and the MDA content and electrolyte leakage were significantly increased. However, Evans blue staining was weaker and the MDA content and EL rate were lower in the transgenic than in the WT plant lines. Under salt stress, the EL of the transgenic lines was 71.85 and 60% that of the WT plants, respectively (Fig. 3c). The MDA contents of the transgenic lines were 81.56 and 73.76% that of the WT plants, respectively (Fig. 3d).

Taken together, these results indicated that *Th2CysPrx* in tobacco improved salt stress tolerance by increasing ROS scavenging and preventing cell damage to maintain better plant growth. The transgenic lines exhibited lower ROS content and less damage than the WT plants, suggesting that *Th2CysPrx* directly affected ROS scavenging and cell protection during NaCl treatment.

### Use of transient overexpression of *Th2CysPrx* in *T. hispida* to further evaluate the results in transgenic tobacco

**Fig. 4:**
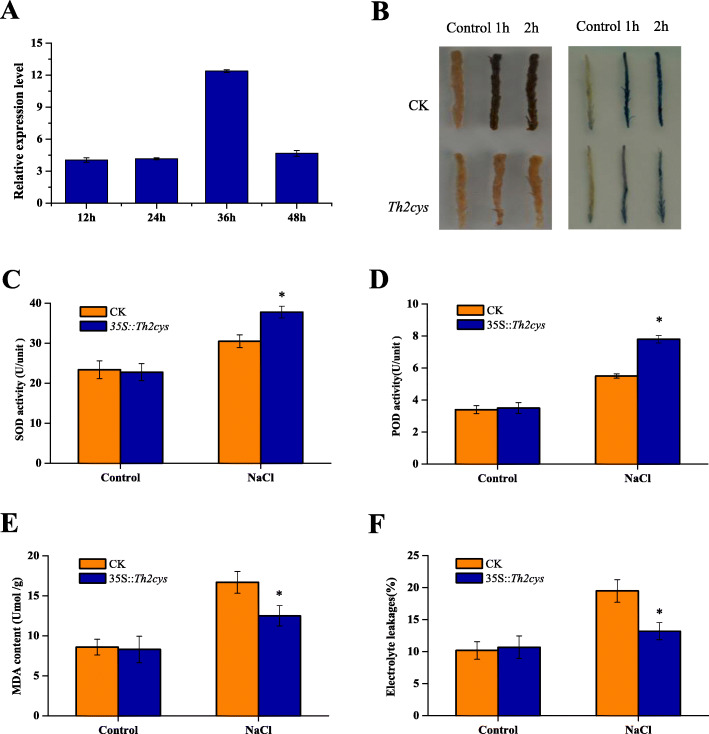
Physiological index analysis of 1-month-old seedlings of CK, *35S::Th2cys* under 150 mM NaCl stress. **a** The relative expression levels of *Th2CysPrx* as measured by qRT-PCR. The data was processed using the 2^-ΔΔCT^ method. **b** DAB and NBT staining. **c** SOD activity levels. **d** POD activity levels. **e** MDA content. **f** Electrolyte leakages. CK: *T. hispida* plants transformed with empty pROKII; *35::Th2cys*: *T. hispida* overexpressing *Th2CysPrx*; All experiments were repeated three times. Data are the mean ± SD of three independent experiments

To confirm the results of heterologous expression in tobacco, *Th2CysPrx* was transiently transferred into *T. hispida*. qRT-PCR analysis showed that the expression of the *Th2CysPrx* gene in *35S::Th2cys* overexpression *T. hispida* was 12.38-fold that of the control, indicating that the transient overexpression line of *T. hispida* was successfully obtained (Fig. 4a). Then, biochemical staining and related physiological indexes were analysed and compared between *35S::Th2cys* and CK plants. The DAB and NBT staining results showed no significant difference between *35S::Th2cys* and CK plants prior to salt stress. However, CK plants showed darker staining than *35S::Th2cys* plants after salt stress, with darker staining after 2 h than 1 h (Fig. 4b).

Similarly, the SOD and POD activity levels also showed no difference between *35S::Th2cys* and CK plants under normal conditions. Under salt stress, SOD activity in CK plants was 80.69% of that in *35S::Th2cys* plants (Fig. 4c), and POD activity in CK plants was 70.51% of that in *35S::Th2cys* plants (Fig. 4d). Under normal conditions, there was no significant difference in terms of MDA or electrolyte leakage content between *35S::Th2cys* and CK plants. However, *35S::Th2cys* plants showed significantly lower MDA level and electrolyte leakage than CK plants, and the MDA content and electrolyte leakage in *35S::Th2cys* plants was 74.85 and 67.70% of that in CK plants (Fig. 4e, f). Altogether, these results showed that *Th2CysPrx* conferred salt tolerance to the transgenic tobacco and *T. hispida* plants.

**Fig. 5:**
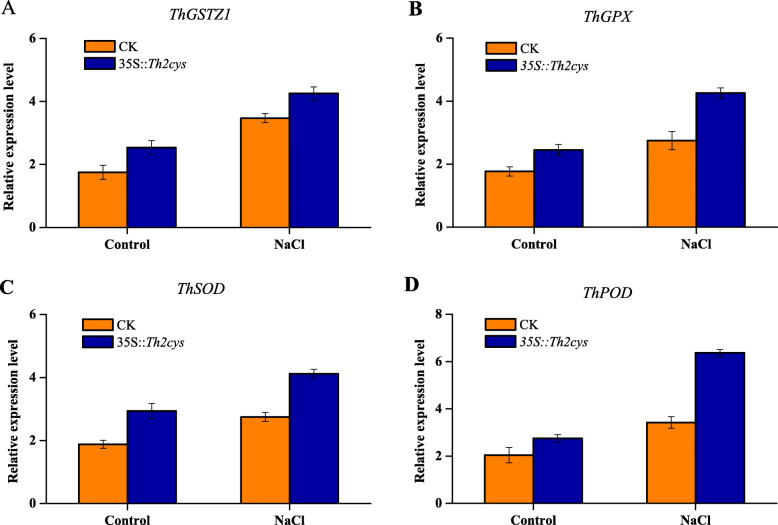
The relative expression levels of *ThGSTZ1*, *ThGPX*, *ThSOD*, *ThPOD* in WT and *35::Th2CysPrx* exposed to NaCl stress. **a** The relative expression levels of *ThGSTZ1*. **b** The relative expression levels of *ThGPX*. **c** The relative expression levels of *ThSOD*. **d** The relative expression levels of *ThPOD*. CK: *T. hispida* plants transformed with empty pROKII; *35::Th2cys*: *T. hispida* overexpressing *Th2CysPrx*; The error bars were calculated from three independent replicates of the qRT-PCR

Additionally, the expression levels of four antioxidant genes (*ThGSTZ1*, *ThGPX*, *ThSOD* and *ThPOD*) in transiently transfected *Th2CysPrx T. hispida* were analysed by qRT-PCR. The results showed that these genes shared similar expression profiles with *Th2CysPrx* in transiently transfected *T. hispida* seedlings, all of which were upregulated after NaCl stress. The expression levels of these genes were 1.22, 1.55, 1.50, and 1.86-fold that of CK plants, respectively (Fig. 5). These results indicated that *Th2CysPrx* overexpression affected *ThGSTZ1*, *ThGPX*, *ThSOD* and *ThPOD* gene expression. Thus, these results indicated that the expression of *Th2CysPrx* altered the expression of other stress-related genes, suggesting that salt stress tolerance regulation involves a complex network.

## Discussion

Under adversity, plants usually accumulate ROS, which leads to damage to protein synthesis and stability to produce cellular macromolecules and membrane lipids and the generation of oxidative stress [29]. The *2CysPrx* plays an important role in scavenging ROS and regulating signal transduction, acting as a molecular chaperone and DNA damage response. In previous studies, 2CysPrx served as a barrier for H_2_O_2_. Rapeseed 2CysPrx activates chloroplast fructose-1,6-bisphosphatase (FBPase) and participates in the Calvin cycle [30]. Kim et al. [31] Showed that *Arabidopsis* 2CysPrx can protect citrate synthase (CS) from heat-induced aggregation and function as a molecular chaperone. Banerjee et al. [32] found that overexpression of *cyanobacterium Anabaena 2CysPrx* could decrease intracellular ROS levels under abiotic stress to reduce cell damage. Overexpression of *2CysPrx* from mungbean can efficiently eliminate cellular ROS to improve tolerance to MV stress in *Arabidopsis* [33]. However, in the process of preventing DNA damage, some organisms use the ROS pathway, and some plants protect the cells from oxidative damage through the non-ROS pathway. For example, 2CysPrx-induced tobacco gene silencing largely affects the regeneration of ascorbic acid and thus the biosynthesis of abscisic acid (ABA) [17]. Above researches showed the 2CysPrx has different functions, most of which are research on glycogen plants, but little exploration in woody halophytes. Therefore, we need to explore whether *Th2CysPrx* prevents cell damage through the ROS pathway in woody halophyte *T.hispida*.

Vidigal et al. [17] demonstrated that *2CysPrx* was the key to H_2_O_2_ clearance and regulated ABA signalling downstream of ROS genes. Many studies have shown that ABA has important biological functions and is an important signalling molecule under abiotic stresses [34–36]. The expression of genes that participate in the ABA signalling pathway can increase plant abiotic tolerance [37, 38]. In this study, compared with WT plants, transgenic *Th2CysPrx* tobacco exhibited increased germination rates, root lengths and chlorophyll contents under salt stress (Fig. 1). Overexpression of *Th2CysPrx* resulted in better SOD and POD activities, and reduced ROS accumulation. Additionally, the expression levels of the *ThGSTZ1*, *ThGPX*, *ThSOD* and *ThPOD* genes were markedly upregulated in transgenic *T. hispida* under salt stress, which indicated that the *Th2CysPrx* gene could improve salt tolerance by increasing antioxidant enzyme and strengthening ROS scavenging activities, leading to reduced ROS accumulation.

We also found that the activities and expression levels of SOD and POD in CK and transgenic *T. hispida* were not consistent under normal condition. However, the overall trend between the activities and expression levels is consistent under NaCl stress. The results showed the studied *ThSOD* and *ThPOD* gene may be key effective gene among SOD and POD family genes responding to NaCl stress (Figs. 2, 3, 4, 5).

It remains unclear whether the *Th2CysPrx* gene improves plant ROS scavenging by activating the expression of ABA signalling pathway genes. So, in future studies, we will further analyze the salt tolerance mechanism of the *Th2CysPrx* gene in *T.hispida*, while comparing the differences in abiotic stress in plants between *2CysPrx* in tobacco and *T.hispida*.

## Conclusion

*Prxs* have an important place in plant growth and development, as well as in responses to stress. However, there is no direct evidence to demonstrate that the *Prxs* gene of *T. hispida* is involved in ROS scavenging and abiotic stress responses. In the present study, a *Th2CysPrx* was isolated from *T. hispida*, and transgenic tobacco and *T. hispida* showed advantages with respect to morphological, physiological, and biochemical traits under salt stress. Additionally, the expression levels of four antioxidant genes (*ThGSTZ1*, *ThGPX*, *ThSOD* and *ThPOD*) were significantly higher in overexpressed *Th2CysPrx* of *T. hispida* than in the control. Altogether, the results indicated that *Th2CysPrx* increased salt tolerance via increasing the expression and activity of antioxidant enzymes and improved ROS scavenging ability. Future studies should assess whether *Th2CysPrx* participates in the ABA signalling pathway.

## Methods

### Plant materials and growth conditions

Tobacco seeds (Longjiang 911) were obtained from the Tobacco Science Research Institute of Heilongjiang Province [39]. And kept in our laboratory. Tobacco seeds stored at 4 °C for 3–5 days were sterilized with 70% (w:v) ethanol for 1 min followed by 3% sodium hypochlorite for 5 min, rinsed eight times with sterile water, and sown onto plates containing half-strength Murashige and Skoog (1/2 MS) medium. They were then placed in a cabinet with a controlled environment (16 h light: 8 h dark) at 22 °C. One week later, the seedlings were transplanted to pots containing a mixture of perlite and soil (1:3 v*/*v) and grown in a greenhouse (14 h light: 10 h dark) at 24 °C with 70% relative humidity.

*T. hispida* seeds (the Turpan Desert Botanical Garden, Xinjiang, 293 China) was planted in a greenhouse in Harbin (China). Seeds for propagation of plant material were harvested from these *T. hispida* plants, and planted on 1/2 MS medium and grown in a greenhouse (14 h light: 10 h dark) at 24 °C with 70% relative humidity and a photon flux density of 250 μE m^− 2^ s^− 1^.

### Generation of transgenic plants

The ORF of *Th2CysPrx* was amplified and cloned into pROKII vector (referred to as *35S::Th2cys*). The primers are shown in Table 1. The *35S::Th2cys* was introduced into *Agrobacterium tumefaciens* EHA105 by electroporation, and the transgenic tobacco was further obtained by the agrobacterium-mediated leaf disc transformation method [40]. Fifteen lines were generated in the T_0_ generation. The two T_3_ homozygous transgenic lines (Line 7 and Line 11) were randomly selected for further analysis.

**Table 1:** List of primers and their applications

Gene symbol	Forward Primers (5′-3′)
pROKII*-Th2CysPrx-*F	ATCG TCTAGAATGGCGTGCGCAGCCCCAACT
pROKII*-Th2CysPrx-*R	AGCTGAGCTGTTAAATTGCAGCGAAGTACTC
*Th2CysPrx-F*	TGAGATCACTGCTTTCAGT
*Th2CysPrx-R*	TGATAACCAATCCTCTGAG
Actin-F	AAACAATGGCTGATGCTG
Actin-R	ACAATACCGTGCTCAATAGG
α-tubulin-F	CACCCACCGTTGTTCCAG
α-tubulin-R	ACCGTCGTCATCTTCACC
β-tubulin-F	GGAAGCCATAGAAAGACC
β-tubulin-R	CAACAAATGTGGGATGCT

Concurrently, *Th2CysPrx* was also transiently overexpressed in *T. hispida*. Specifically, it was transiently transformed into one-month-old *T. hispida* seedlings with *35S::Th2cys* to overexpress *Th2CysPrx* and with empty pROKII plasmid (used as a control, CK) according to Zheng et al. [41] with some modifications*.* In particular, single colonies of *A. tumefaciens* strain EHA105 harbouring *35S::Th2cys* or empty pROKII were cultured to an OD_600_ = 0.8, and the cells were harvested by centrifugation. The transformants used [1/2 MS + 3% (w/v) sucrose + 150 μM acetosyringone + 0.01% (w/v) Tween 20] were adjusted to an OD_600_ = 0.8. *T. hispida* was immersed in transformation solution and incubated at 25 °C for 4 h. Subsequently, 2% sucrose was used to quickly wash the seedlings once for 1 min, after which they were planted vertically on 1/2 MS solid medium for 12, 24, 36 or 48 h. Total RNA was isolated from every samples using the CTAB method [42], and 1 mg of RNA was reverse transcribed with the PrimeScript™ RT reagent Kit (TaKaRa, China). The resulting cDNA product was diluted to 10x and used as a template for the qRT-PCR analyses. Real-time RT-PCR was conducted using a Bio-Rad (MJ) Opticon™^2^ System (Bio-Rad, Hercules, USA) Actin (FJ618517), α-tubulin (FJ618518), and β-tubulin (FJ618519) were used as internal controls to normalize the amount of total RNA present in each reaction. The primers used are listed in Table 1. The PCR conditions were 94 °C for 30 s, 45 cycles of 94 °C for 12 s, 58 °C for 30 s, 72 °C for 40 s, and 80 °C for 1 s for plate reading. A melting curve was generated for each sample at the end of each run to assess the purity of the amplified products. To determine the expression of *Th2CysPrx* and several antioxidant genes. The primers are listed in Table 1. The reaction system and procedure for qRT-PCR were performed according to Gao et al. [28]. The 2^-ΔΔCt^ method was used to calculate the relative gene expression [43].

### Analysis of stress tolerance

The T_3_ generation seeds of *Th2CysPrx* transgenic tobacco were surface sterilized and sown on 1/2 MS agar medium or 1/2 MS with 125 mM NaCl. Germination was recorded after 10 d. In addition, 3-day-old seedlings sown on 1/2 MS were transferred to 1/2 MS medium or 1/2 MS with 125 mM NaCl for 2 weeks to compare the fresh weight and root length between transgenic and wild type (WT) lines.

### Physiological analysis

Seven-day-old tobacco seedlings sown on 1/2 MS were transferred to a mixture of perlite and soil (1:3 v/v). After 6 weeks, the tobacco seedling roots were watered with the solution of 200 mM NaCl. Simultaneously, the seedlings were watered with water as a control. After 7 d, the leaves of stressed and control tobacco were harvested.

One-month-old seedlings of the transient transgenic *T. hispida* seedlings (*35S::Th2cys* and CK) exposed to 150 mM NaCl for 12 h were harvested, and the physiological index was measured, respectively. The SOD, POD activities and MDA contents were measured according to Wang et al. [44]. The EL was measured according to Ben-Amor et al. [45]. The chlorophyll contents were measured following the method of Lichtenthaler [46].

### Detection of ROS and cell death

The transgenic and WT/control young leaves were collected after 0, 1 or 2 h of NaCl treatment, and histochemical staining analysis was carried out immediately. To detect superoxide accumulation, hydrogen peroxide accumulation, and cell death, leaves were infiltrated with DAB, NBT and Evans blue staining according to the method described in detail by Zhang et al. [47] and Kim et al. [48]. Evaluation of ROS production in intact guard cells was performed by staining with H_2_DCF-DA (Sigma-Aldrich) [49].

### Statistical analysis

Each experiment was repeated at least three times independently. Error bars represent standard deviations. Differences were compared using Student’s t-test. *P* < 0.05 was considered significant, which is indicated by *.

## Supplementary information

**Additional file 1 Figure S1**. The relative expression levels of *Th2CysPrx* gene in the WT and transgenic tobacco as measured by qRT-PCR. The data was processed using the 2^-ΔΔCT^ method. WT: the wild type tobacco. Line1–14: different transgenic tobacco lines; All experiments were repeated three times. The error bars represent the standard deviation.

## Data Availability

All data and materials generated or analyzed during this study are included in this article or are available from the corresponding author on reasonable request.

## Abbreviations

SOD: Superoxide dismutase
POD: Peroxidase
ROS: Reactive oxygen species
*CAT*: *Catalase*
*GPX*: *Glutathione peroxidase*
*GST*: *Glutathione S-transferases*
MV: Methyl viologen
*P5CS*: Δ1-pyrroline-5-carboxylate synthetase
Prxs: Peroxiredoxins
H_2_O_2_: Hydrogen peroxide
qRT-PCR: Real-time quantitative PCR
WT: Wild-type
DAB: 3,3′-Diaminobenzidine
NBT: Nitro blue tetrazolium
H_2_DCF-DA: 2,7-Dichlorofluorescin diacetate
MDA: Malondialdehyde
EL: Electrolyte leakage
FBPase: Fructose-1,6-bisphosphatase
CS: Citrate synthase
ABA: Abscisic acid
1/2 MS: Half-strength murashige and skoog
