# Correction to: A 2-*Cys peroxiredoxin* gene from *Tamarix hispida* improved salt stress tolerance in plants

**DOI:** 10.1186/s12870-020-02600-3

**Published:** 2020-08-19

**Authors:** Yuanyuan Wang, Zhongyuan Liu, Peilong Wang, Bo Jiang, Xiaojin Lei, Jing Wu, Wenfang Dong, Caiqiu Gao

**Affiliations:** State Key Laboratory of Tree Genetics and Breeding (Northeast Forestry University), Harbin, 150040 China

**Correction to: BMC Plant Biology 20, 360 (2020)**

**https://doi.org/10.1186/s12870-020-02562-6**

In the original publication of this article [1], the author indicates two errors in the article.

1. “CK: Tobacco plants transformed with empty pROKII; 35::Th2Cys: Tobacco overexpressing Th2CysPrx;” in the legend of Fig. 3 should be removed.
2. In the sentence “Fifteen lines were generated in the T0 generation.”, “Fifteen” should be “Fourteen”.
